# Epidemiology and outcomes of COVID-19 in HIV-infected individuals: a systematic review and meta-analysis

**DOI:** 10.1038/s41598-021-85359-3

**Published:** 2021-03-18

**Authors:** Paddy Ssentongo, Emily S. Heilbrunn, Anna E. Ssentongo, Shailesh Advani, Vernon M. Chinchilli, Jonathan J. Nunez, Ping Du

**Affiliations:** 1 Department of Public Health Sciences, Penn State College of Medicine and Milton S. Hershey Medical Center, Hershey, PA USA; 2 Center for Neural Engineering, Department of Engineering, Science and Mechanics, The Pennsylvania State University, University Park, PA USA; 3 Division of Trauma Surgery, Department of Surgery, Penn State College of Medicine and Milton S. Hershey Medical Center, Hershey, PA USA; 4 Department of Oncology, Georgetown University School of Medicine, Georgetown University, Washington, DC USA; 5 Terasaki Institute of Biomedical Innovation, Los Angeles, CA USA; 6 Department of Medicine, Penn State College of Medicine and Milton S. Hershey Medical Center, Hershey, PA USA

**Keywords:** Epidemiology, Viral infection

## Abstract

Susceptibility to severe acute respiratory syndrome coronavirus 2 (SARS-CoV-2) infection and the risk of mortality among people living with human immunodeficiency virus (HIV)/acquired immunodeficiency syndrome (AIDS) (PLWHA) is largely unknown. PLWHA are unique due to their altered immune system from their history of chronic HIV infection and their use of antiretroviral therapy, some of which have been used experimentally to treat coronavirus disease 2019 (COVID-19). Therefore, we conducted a systematic review and meta-analysis to assess the epidemiology of SARS-COV-2/HIV coinfection and estimate associated mortality from COVID-19 (Prospero Registration ID: CRD42020187980). PubMed, SCOPUS, OVID and Cochrane Library databases, and medRxiv preprint repositories were searched from January 1, 2020, to December 12, 2020. Data were extracted from studies reporting COVID-19 attack and mortality rates in PLWHA compared to their HIV-negative counterparts. Pooled attack and mortality risks were quantified using random-effects models. We identified 22 studies that included 20,982,498 participants across North America, Africa, Europe, and Asia. The median age was 56 years, and 50% were male. HIV-positive persons had a significantly higher risk of SARS-CoV-2 infection [risk ratio (RR) 1.24, 95% CI 1.05–1.46)] and mortality from COVID-19 (RR 1.78, 95% CI 1.21–2.60) than HIV-negative individuals. The beneficial effects of tenofovir and protease-inhibitors in reducing the risk of SARS-CoV-2 infection and death from COVID-19 in PLWHA remain inconclusive. HIV remains a significant risk factor for acquiring SARS-CoV-2 infection and is associated with a higher risk of mortality from COVID-19. In support of the current Centers for Disease Control and Prevention (CDC) guidelines, persons with HIV need priority consideration for the SARS-CoV-2 vaccine.

## Introduction

As of January 30, 2021, the number of confirmed cases of coronavirus disease 2019 (COVID-19) exceeded 102 million, with more than 2.2 million deaths registered globally^1^. Prior studies have identified that older age or underlying comorbidities such as cancer, diabetes, cardiovascular disease, hypertension, heart failure, chronic kidney disease, and obesity increased the risk of severe acute respiratory syndrome coronavirus 2 (SARS-CoV-2) infection and mortality from COVID-19^2–5^. However, a large gap in the literature exists on the impact of human immunodeficiency virus (HIV)/acquired immunodeficiency syndrome (AIDS) on the susceptibility and severity of infection with SARS-CoV-2.

There is a growing concern that the immunosuppressing nature of HIV^4^ may make people living with HIV/AIDS (PLWHA) more susceptible to SARS-CoV-2 infection and more likely to present with severe COVID-19 when infected. Mounting evidence shows PLWHA with low CD4 counts as well as those not on antiretroviral (ARV) therapy have the greatest risk of contracting severe symptoms of COVID-19^6,7^. However, other studies suggest that immunosuppression and low CD4 cell counts might protect PLWHA from developing the cytokine storm observed in patients with COVID-19^8^. With nearly 40 million PLWHA across the globe, it remains urgent to characterize the epidemiology and outcomes of COVID-19 such as intensive care unit (ICU) admission, mechanical ventilation, and death among this group^9^.

To address this knowledge gap, we conducted a systematic review and meta-analysis of the literature to (1) assess the risk of SARS-CoV-2 infection among PLWHA and (2) estimate the mortality risk from COVID-19 for PLWHA.

## Methods

### Information source, search strategy, and study selection

The present study has been registered with PROSPERO (registration ID: CRD42020187980) and is reported per Preferred Reporting Items for Systematic Reviews and Meta-Analyses (PRISMA) statement (Supplemental Table 1)^10^. We searched PubMed, Scopus, OVID, Web of Science, and Cochrane Library from January 1, 2020 to December 12, 2020. In addition, we searched grey literature using Google Scholar and Medrxiv servers to identify any preprints or associated publications. Furthermore, we performed hand-searching of the reference lists of included studies, relevant reviews, or other relevant documents. Studies reporting susceptibility and death from COVID-19 in people with and without HIV infections were included in our search and analysis. We did not limit the search to study design, country of publication, or language to provide a comprehensive landscape of the COVID-19 infection. Articles published in other languages were appropriately translated and further included/excluded during the screening process.

The primary outcome was susceptibility to SARS-CoV-2 among PLWHA compared to their HIV-negative counterparts. A secondary outcome was the mortality risk of COVID-19 patients with HIV compared to COVID-19 patients without HIV. Predefined search terms determined by the Medical Subject Headings (MeSH) included multiple combinations of the following: “Human Immunodeficiency Virus” OR “HIV” OR “AIDS”, AND “COVID-19” OR “Coronavirus.” Studies found as a result of our initial search were transferred into Endnote, which further removed duplicate studies.

### Eligibility criteria

Studies were selected according to the following criteria: participants, condition or outcome(s) of interest, study design and context.

1. *Participants (population)* We included studies involving individuals with and without HIV tested for SARS-CoV-2, regardless of age, country, or antiretroviral therapy.
2. *Condition or outcome(s) of interest* The primary outcome was the susceptibility to SARS-CoV-2 among PLWHA compared to their non-HIV counterparts. The secondary outcome was mortality risk of COVID-19 for PLWHA in comparison to COVID-19 patients without HIV/AIDS.
3. *Study design and context* Eligible studies were randomized controlled trials, observational cohort (prospective or retrospective), cases series and case–control studies. We excluded case reports. Criteria of inclusion included articles that reported the risk ratio (RR) of COVID-19 infection and severity in PWLHA compared to those without HIV, or if a study provided enough information to calculate the RR of intensive care unit (ICU) admission, mechanical ventilation and death from COVID-19 among PLWHA compared to HIV-negative COVID-19 patients.

### Data extraction

Two investigators (ESH and AES) individually screened all titles and abstracts against the inclusion criteria. Articles were coded as “yes” or “no” for inclusion or exclusion. If both reviewers coded an article as “yes”, it was included for full-text review, if both reviewers coded as “no”, it was removed from the further screening process, and if there was a discrepancy, then decision was reached through mutual consensus with a third investigator (PS). After initially screening articles for inclusion based on titles and abstracts, ESH and AES then screened full-text articles. Disagreements were resolved by discussion to meet a consensus, if necessary. In scenarios where consensus was not reached, disagreements were resolved by a third investigator (PS).

We extracted the following information: year of publication, date of the study, sample size, rates of COVID-19 in HIV-positive and negative individuals, mortality rates, proportion on mechanical ventilators and ICU admissions. Relevant effect size estimates, such as the relative risk, odds ratios or hazard ratios of mechanical ventilation, ICU admission, and death were extracted as well.

### Study quality assessment

Two investigators (ESH and AES) independently assessed the quality of the included studies. The Newcastle–Ottawa Scale (NOS) was utilized for the quality assessment of the included studies^11^. NOS scale rates observational studies based on 3 parameters: selection, comparability between the exposed and unexposed groups, and exposure/outcome assessment. It assigns a maximum of 4 stars for selection, 2 stars for comparability, and 3 stars for exposure/outcome assessment. Studies with less than 5 stars were considered low quality, 5 to 7 stars moderate quality, and more than 7 stars high quality.

### Data synthesis and statistical analysis

A narrative approach was used to describe the number of studies, study settings, diagnostic criteria COVID-19 (laboratory-confirmed by polymerase chain reaction (PCR) of SARS-CoV-2 RNA or clinical diagnosis), ARV therapy such as nucleos(t)ide reverse transcriptase inhibitors (NRTI), (e.g., tenofovir disoproxil fumarate, tenofovir alafenamide, emtricitabine, lamivudine), non-nucleoside reverse transcriptase inhibitors (NRTI), (e.g., doravirine); protease-inhibitors (e.g., darunavir, lopinavir or ritonavir), integrase inhibitors (e.g., dolutegravir), CD4 count, HIV viral load, pre-existing comorbidities (i.e. cancer, diabetes, cardiovascular disease, hypertension, heart failure, chronic kidney disease, chronic liver disease, asthma, and obesity) and study-level patient demographics.

The *metaprop* and *metagen* functions from the R package *meta* were used to calculate the pooled effect estimates using random-effects models^12^. DerSimonian and Laird random-effects method was applied to estimate the pooled between-study variance (heterogeneity)^13^. RR of SARS-CoV-2 among PLWHA compared to HIV-negative individuals were estimated. Also, estimated were RRs for COVID-19 mortality, ICU admissions, and use of ventilation. Individual and pooled estimates were graphically displayed using forest plots. Between-study heterogeneity was assessed with *I*^2^ statistics, expressed as percent low (25%), moderate (50%), and high (75%) and Cochrane’s *Q* statistic (significance level < 0.05)^14^. To investigate the sources of heterogeneity, subgroup analysis was performed by country of the study. Potential ascertainment bias was assessed qualitatively with funnel plots, by plotting the study effect size against standard errors of the effect size, and quantitatively with the Egger’s test^15^. Results were reported as RR or prevalence expressed as a percentage. All statistical analyses were performed with R software, version 3.4.3 (R, College Station, TX).

## Results

### Overview

**Figure 1:**
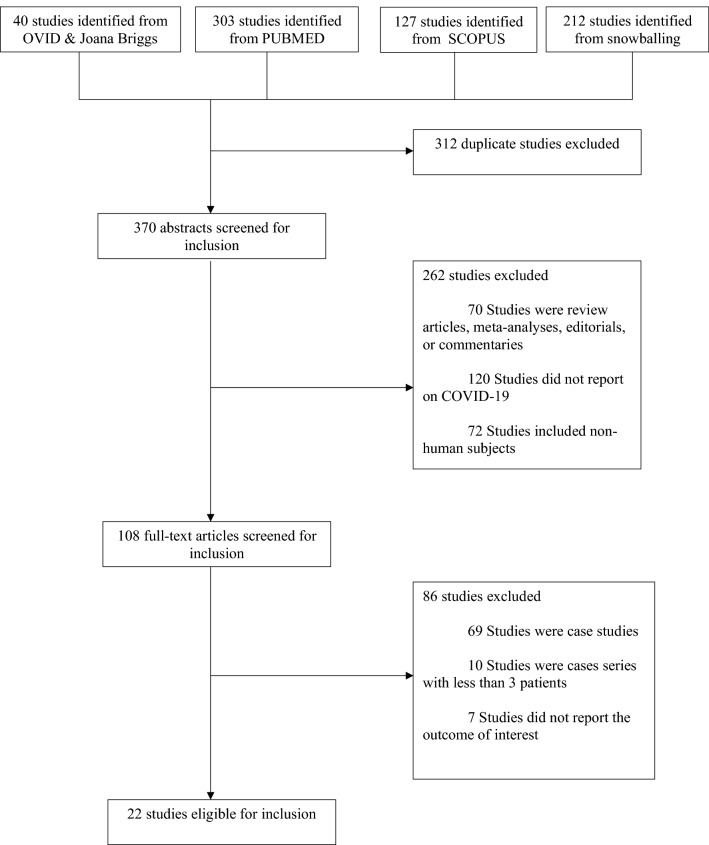
PRISMA flowchart of a systematic review and meta-analysis of HIV and SARS-CoV-2 coinfection.

As seen in Fig. 1, a total of 682 studies were identified from four databases. 312 were determined to be duplicates, leaving 370 for the initial screening of titles and abstracts. 262 were then excluded based on the abstract. Of the 108 full-text articles screened, an additional 86 were excluded, leaving a total of 22 studies included for the analysis. These studies represented North America, Africa, Europe, and Asia. The articles that were ultimately included contained a total of 20,982,498 sample size. Table 1 summarizes the study-level sociodemographic variables of the study sample and diagnostic criteria for COVID-19 (clinical and laboratory-confirmed by PCR). The median age of the patients included in the study was 56 years. On average, 66.0% of the participants were male. The most common comorbidities in the HIV-positive population were hypertension, diabetes, chronic obstructive pulmonary disease and chronic kidney disease. Overall, the median CD4 count was 538 cells/μL. Over 96% of PLWHA were on ARV therapy, and over 80% of the HIV-positive patients were virally suppressed (than 50 copies of HIV/mL). All studies except one reported diagnosis of COVID-19 with SARS-CoV-2 RNA reverse transcription PCR (RT-PCR) of nasopharyngeal or throat swabs.

**Table 1:** Summarized study-level patient sociodemographic characteristics.

Author	Country	Sample size	Age (y)	% Male	# HIV	% HIV	% on ART	CD4 T-cell/μL (median)	HIV Viral load	Study quality score	Comorbidities in HIV population	COVID-19 diagnosis	ART effects on susceptibility/deaths
Härter et al.^16^	Germany	33	48	90	33	100	100	670 (69–1715)	HIV RNA < 50 copies/mL (30/32) 2/32 had detectable viremia, all needed ventilation, and 1 died	7	HTN(10/33), COPD (6/33), diabetes (4/33), CVD (3/33), CKF (2/33)	SARS-CoV-2 RNA RT-PCR from nasopharyngeal, swabs in 29, bronchoalveolar lavage or sputum in 2 cases; in two cases, information not available in 2 cases	NRTIs (31/33) INSTI (20/33) PI (4/33) Non-NRTI (9/33) NRTIs TAF (16/31) TDF (6/31) FTC (22/31) 3TC (9/31) Died (3) BIC/TAF/FTC (33%) DOR/TDF/FTC(33%) DRV/RTV/RGV(33%)
Blanco et al. ^17^	Spain	5	38	60	5	100	100	564	HIV RNA < 50 copies/mL (4/5)	7	Asthma (1/5) Hypothyroidism (1/5)	SARS-CoV-2 RNA RT-PCR from nasopharyngeal swabs in all patients	TAF/FTC (2/5) ABC/3TC (2/5) No ART (1/5) No death recorded
Gervason et al.^18^	Italy	47	51	77	47	100	100	636	HIV RNA < 50 copies/mL (44/47)	7	HTN(14/47), COPD (2/47), diabetes (3/47), CVD (2/47), CKF (4/47), cancer (3/47), epilepsy (2/47), Hep B or C (5/47)	SARS-CoV-2 RT-PCR (26) IgG/IgM rapid test (2) Clinical symptoms (19)	Pre-hospitalization ART not provided
Richardson et al.^19^	United States	5,700	63	60	43	0.75	–	–	–	8	–	SARS-CoV-2 RNA RT-PCR of nasopharyngeal swabs	–
Chen et al.^20^	China	203	54	53	2	0.99	–	–		8	–	Sputum and throat swab SARS-CoV-2 RNA RT-PCR	–
Karmen-Tuohy et al.^21^	United States	63	60 HIV+ 61 HIV −	91 HIV+ 91 HIV−	21	33	100	298	HIV RNA < 50 copies/mL (1/21)	8	HTN(7/21), COPD (4/21), diabetes (4/21), CVD (1/21), CKF (4/21), cancer (3/21), hyperlipidemia(4/21)	SARS-CoV-2 RT-PCR	Pre-hospitalization ART not provided
Bhatraju et al.^22^	United States	24	64	63	1	4	–	–	–	8	–	SARS-CoV-2 RT-PCR	Pre-hospitalization ART not provided
Cummings et al.^23^	United States	257	62	67	8	3	–	–	–	8	–	SARS-CoV-2 PCR	Pre-hospitalization ART not provided
Prieto-Alhambra et al.^24^	Spain	121,263	–	42	311	0.26	–	–	–	8	–	SARS-CoV-2 RT-PCR and/or a clinical diagnosis	Pre-hospitalization ART not provided
Argenziano et al.^25^	United States	1000	63	60	21	2	–	–	–	8	–	SARS-CoV-2 RT-PCR	Pre-hospitalization ART not provided
Marcello et al.^26^	United States	13,442	53	56	159	1	–	–	–	8	–	Real-time RT-PCR assays of nasopharyngeal swabs	Pre-hospitalization ART not provided
Crotty et al.^27^	United States	289	59	–	7	2	–	–	–	8	–	RT-PCR a nasopharyngeal	Pre-hospitalization ART not provided
Suwanwongse and Shabarek et al.^28^	United States	9	58	78	9	100	89	617	HIV RNA < 50 copies/mL (8/9)	7	HTN(6/9), COPD (4/9), Diabetes (3/9),HCV(3/9)	RT-PCR a nasopharyngeal	Only 2 of the 9 patients survived. One was on FTC, TAF, DTG, RTV, DRV and the other on EVG, FTC, TAF, cobicistat. Of the 7 that died, 2 were on TDF containing regimen and 4 on TAF containing regimen
Vizcarra et al.^29^	Spain	51	53.3	84	51	100	100	565 (296–782)	Last HIV-RNA < 50 copies per mL, N = 50 /51(98%)	7	HTN (18/51), diabetes (7/51), CKF (6/51), CLD(24/51)	SARS-CoV-2 RT-PCR and/or a clinical diagnosis	The 51 HIV individuals with COVID-19 37 (73%) were on Tenofovir (TAF or TDF) compared to 487 (38%) HIV-infected individuals not with COVID-19. Protease inhibitors, NNRTI frequencies were similar between the two groups
Bhaskaran et al.^30^	United Kingdom	17.3 million	48 HIV+ 49 HIV−	65 HIV+ 50 HIV−	27,480	0.2	–	–	–	9	HTN(19%), CLD (3.4%), CVD (5.3%), stroke (2.0%), chronic respiratory disease (4.0%)	–	Data on antiretroviral therapy use not reported
Boulle et al.^31^	South Africa	3.4 million	> 20 years	37	536,574	16	–	–		8	–	SARS-CoV-2 RT-PCR test	Twofold association of COVID-19 death with HIV irrespective of viremia or immunosuppression before the COVID-19 episode. TDF was associated with lower COVID-19 mortality compared to other antiretrovirals
Charre et al.^32^	France	19,113	47	41	77	0.4	99	529	HIV-RNA < 50 copies per mL, N = 69 (89.6%)	7	–	SARS-CoV-2 RT-PCR test	All HIV-infected patients TDF/TAF, N = 52 (67.5%); NNRTI, N = 23 (29.9%); INSTI, N = 48 (62.3%); boosted PI, N = 9 (11.7%) HIV-SARS-CoV-2 coinfected patients: 10/12 (83%)
D'Souza et al.^33^	United States	3411	57	46	2078	61	92	682	HIV-RNA < 50 copies per mL, N = 69 (74%)	8	–	All diagnosis are assumed to be PCR-based	Data on antiretroviral therapy use not reported
Geretti et al.^34^	United Kingdom	47,592	56 HIV+ 74 HIV−	66 HIV+ 57 HIV−	122	0.26	92	–	–	8	Cardiac disease (20/117), COPD (13/120), diabetes (28/117), CKF (21/116), cancer (4/118), obesity (19/112) malnutrition (5/112)	SARS-CoV-2 RNA PCR	Data on antiretroviral therapy use not reported
Huang et al.^7^	China	6001	37	90	6001	100	92	–	HIV-RNA < 20 copies per mL, 3334/5004 (66.63)	8	–	Confirmed cases (by RT-PCR) and clinically diagnosed cases	All PLWHA NRTI = 92% NNRTI = 81% PI = 8% PLWHA and COVID-19 NRTI = 91% NNRTI = 81% PI = 3% No statistical difference
Tesorieret et al.^6^	United States	2988	–	–	2988	100	–	–		8	–	PCR confirmed SARS-CoV-2 infection	Data on antiretroviral therapy use not reported
del Amo et al.^35^	Spain	236 (HIV with COVID-19)	–	236	236	100	100	–	–	8	–	SARS-CoV-2 PCR test in all 236 patients with COVID-19	All PLWHA: TAF/FTC (33%),ABC/3TC (26%) and TDF/FTC (16%) PLWHA with COVID-19: TAF/FTC (42%),ABC/3TC (24%) and TDF/FTC (9%)

ART: antiretroviral therapy; NRTIs: nucleos(t)ide reverse transcriptase inhibitors; NNRTI non-nucleoside reverse transcriptase inhibitors; TDF: tenofovir disoproxil fumarate; TAF: tenofovir alafenamide; FTC: emtricitabine; DOR: doravirine; DRV: darunavir; DTG: dolutegravir; 3TC: lamivudine; PI: protease-inhibitor; HTN: hypertension; CKD: chronic kidney disease. PCR: polymerase chain reaction; Hep: Hepatitis; HCV: Hepatitis C virus; RT-PCR Reverse transcription Polymerase Chain Reaction; CLD: Chronic Liver Disease; NSTI, integrase strand transfer inhibitor;

PLWHA; persons living with HIV/AIDS.

### Estimated risk of HIV on SARS-CoV-2 susceptibility

**Figure 2:**
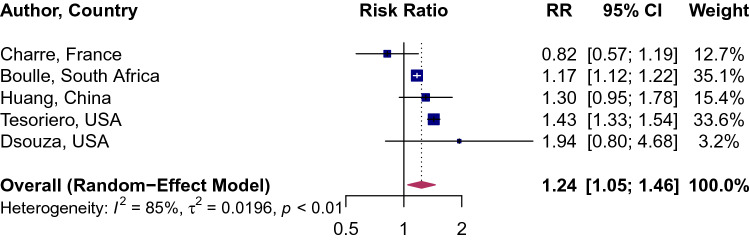
Association of HIV and attack rate of SARS-CoV-2. Blue squares and their corresponding lines are the point estimates and 95% confidence intervals from each study. Maroon diamond represents the pooled effect estimate.

**Figure 3:**
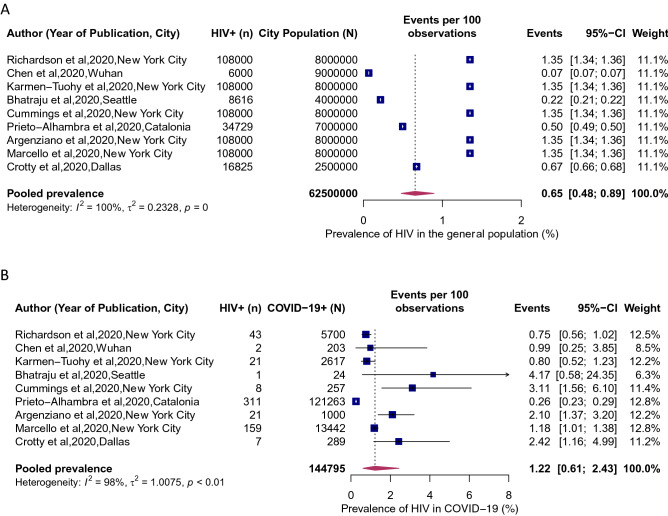
HIV prevalence in populations COVID-19 and the general population. (**A**) Local Prevalence of HIV in cities where COVID-19 studies were conducted. (**B**) Prevalence of HIV in patients hospitalized for COVID-19. Blue squares and their corresponding lines are the point estimates and 95% confidence intervals per each study. Maroon diamond represents the pooled effect estimate.

**Figure 4:**
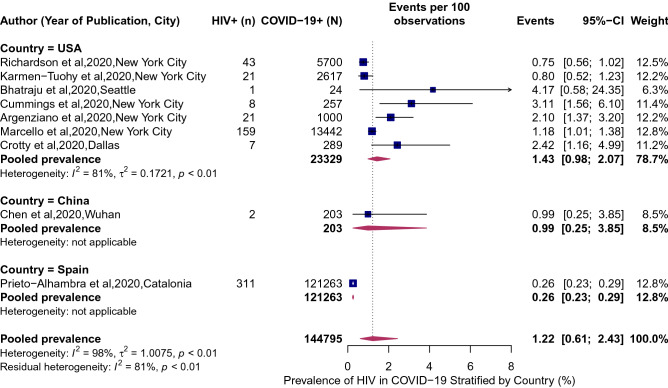
Prevalence of HIV in patients hospitalized for COVID-19 stratified by country. Blue squares and their corresponding lines are the point estimates and 95% confidence intervals per each study. Maroon diamond represents the pooled effect estimate.

HIV was associated with a significantly higher risk of SARS-CoV-2 infection (RR 1.24, 95% CI 1.05–1.46; Fig. 2). Between-study variation was high (*I*^2^ = 85, *p* = 0.0003). The pooled prevalence of HIV in COVID-19 patients was 1.22% (95% CI 0.61–2.43%; Fig. 3b; *I*^2^ = 98%; p < 0·01). The prevalence of HIV in COVID-19 patients ranged from a low of 0.26% (95% CI 0.23–0.29%) in Catalonia, Spain to a high of 4.17% (95% CI 0.58–24.35%) in Seattle, USA. The point estimates for the prevalence of HIV of the general population in the analyzed cities was half the HIV prevalence in among COVID-19 patients: 0.65% (95% CI 0.48–0.89%; Fig. 3a). When we stratified the analysis by the country (Fig. 4), we found a noticeable difference in the pooled HIV prevalence among COVID-19 patients in the United Sates (1.43%, 95% CI 0.98–2.07%) compared to Spain (0.26%, 95% CI 0.23–0.29%) but the difference was not significant compared to the prevalence in China (0.99%, 95% CI 0.25–3.85%).

### Association of HIV/AIDS and COVID-19 clinical outcomes

**Figure 5:**
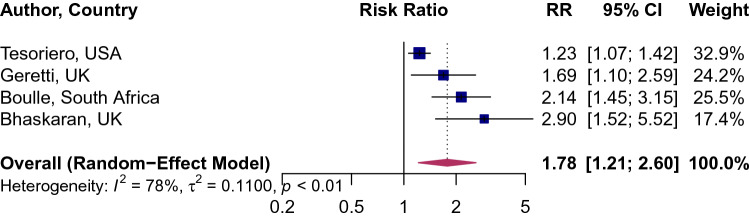
Association of HIV and mortality risk from COVID-19. Blue squares and their corresponding lines are the point estimates and 95% confidence intervals per each study. Maroon diamond represents the pooled effect estimate.

**Figure 6:**
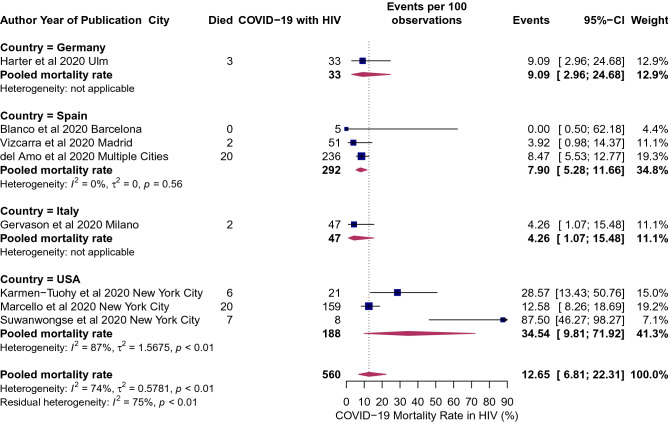
Mortality rates in COVID-19 individuals living with HIV/AIDS: mortality rates in HIV patients hospitalized COVID-19 stratified by country. Blue squares and their corresponding lines are the point estimates and 95% confidence intervals per each study. Maroon diamond represents the pooled effect estimate.

The overall pooled RR of COVID-19 mortality associated with HIV was 1.78 (95% CI 1.21–2.60), implying there is nearly an 80% excess risk of death among HIV patients as compared to the individuals without HIV/AIDS. Between-study variation was moderate (*I*^2^ = 78, p = 0.002; Fig. 5). The pooled mortality rate among HIV positive patients was 12.65% (95% CI 6.81–22.31%, *I*^2^ = 74%; p < 0.01; Fig. 6). The pooled mortality rate ranged from a high of 35% (95% CI 10–72%) in the United States to a low of 4% (95% CI 1.07–15.48%; Fig. 6) in Italy. Tesoriero and colleagues reported that among PLWHA, hospitalization risk increased with disease progression from HIV stage 1 to stage 2 (adjusted RR [aRR] [95% CI] 1.27 [1.09–1.47]) and stage 3 (aRR [95% CI] 1.54 [1.24–1.91]), and for those virally unsuppressed (aRR [95% CI] 1.54 [1.24–1.91])^6^. No association was observed between HIV status and ICU admission among COVID-19 patients (RR: 1.50; 95% CI 0.84–9.2.67; Supplemental Fig. 1).

### ARV therapy types and the risk of COVID-19 diagnosis and severe outcomes

Four studies documented the risk of COVID-19 diagnosis and severity by ARV therapy. In a study by del Almo and colleagues, after stratification by NRTI regimen, persons receiving tenofovir disoproxil fumarate/emtricitabine ( TDF/FTC) had the lowest risk for COVID-19 diagnosis (16.9 per 10,000), hospitalization (10.5 per 10,000), ICU admission (0 per 10,000) and death (0 per 10,000)^35^. However, in the adjusted multivariable logistic regression model, Vizcarra and colleagues found that higher use of tenofovir before the COVID-19 pandemic was associated with nearly fourfold greater risk for the diagnosis of COVID-19 (OR 3.7 [95% CI 1.6–8.7])^29^. Furthermore, Haerter and colleagues did not find clear evidence for a protective effect of tenofovir and boosted darunavir-based ARV therapy^16^. In addition, the results from a France study did not support a protective role of tenofovir against COVID-19^32^. All 5 patients reported in the study by Blanco received boosted-protease inhibitor ARV therapy, with the rationale that HIV protease inhibitors might have activity against the coronavirus protease. By the reporting of their study findings, no patient had died^17^.

### Study quality, publication bias and sensitivity analyses

The median study quality score was 8 out of 9 (range: 7–8; Table 1). Visual inspection of funnel plot shows clear evidence of asymmetry (Supplemental Fig. 2). Egger’s test for asymmetry was significant (p = 0.005), indicative of possible publication bias.

## Discussion

This meta-analysis found that persons living with HIV have a higher risk of SARS-CoV-2 infection and mortality risk from COVID-19 than people without HIV. Furthermore, our estimates suggest the prevalence of HIV in COVID-19 patients and associated mortality vary globally.

Preexisting chronic conditions such as hypertension, diabetes, and cardiovascular diseases are prevalent in PLWHA, and given these comorbidities play a significantly influential role in COVID-19 severity^3^, PLWHA, even on ARV therapy, may still present with a compromised immune system, and subsequently have an increased risk of COVID-19 and associated adverse outcomes. In addition to immunosuppression associated with HIV/AIDS, PLWHA are at risk of anemia, neutropenia, thrombocytopenia, and abnormal serum electrolytes^36^, which also play a critical role in the disease course of COVID-19. Hence, it remains vital for PLWHA to retain regular HIV care and maintain good adherence to ARV therapy.

The mean age of HIV-positive patients with COVID-19 was 55 years, which is a decade younger than the mean age of hospitalized COVID-19 patients in the general population. The substantial difference in age of COVID-19 results from the effect of premature aging of PLWHA due to chronic inflammation associated with HIV infection or high prevalence of certain behavioral risk factors (e.g., smoking). Consequently, the rate of comorbidities that are commonly seen in older, non-HIV populations (such as cardiovascular disease) are prevalent in PLWHA at a much younger age^37^.

PLWHA are generally characterized by a dysregulated immune system. A rapid and well-coordinated innate immune response provides a vital defense system against viral infections^38^. It remains crucial to maintain a balance in the immune system, characterized by a balance in CD4 cell subpopulations that consist of effector-T cells and memory T-cells^39,40^. Among patients with COVID-19, evidence exists of high levels of proinflammatory cytokines and chemokines leading to an increase in the severity of COVID-19 infections, higher consumption of CD4+ and CD8+ T-cells, decrease in regulatory T-cells, and a altered innate immune environment leading to a cytokine storm and worsen damaged tissue^40,41^. In addition, evidence also suggests that subgroups of COVID-19 patients might suffer from hyperinflammatory syndrome termed as secondary hemophagocytic lymphohistiocytosis (sHLH) that is often triggered by underlying viral infections or sepsis and leads to fulminant and fatal hypercytokinaemia with multiorgan failure^41^. Though the incidence of sHLH among patients with HIV might be rare, coinfection of HIV patients with COVID-19 might result in sHLH leading to heightened severity of disease and high mortality.

An important component and determinant of HIV infections involves not only CD4 count but also viral load and access and adherence to ARV therapy, which remains an essential aspect of long-term outcomes, including progression to AIDS and survival among HIV patients^42^. Non-adherence to ARV therapy has shown to be associated with low CD4 count, high viral load, and risk of AIDS and death^43^. Among PLWHA, hospitalization risk increased with disease progression and for those virally unsuppressed^6^. Although some ARV therapies have been proposed to protect against COVID-19, the data remain uncertain. In molecular docking studies, tenofovir has been recently shown to bind to SARS-CoV-2 RNA polymerase (RdRp) with binding energies comparable to those of native nucleotides and to a similar extent as remdesivir^44^. Observational studies have indicated a potential benefit of protease-inhibitors (lopinavir or ritonavir) in COVID-19 by slowing the disease's progression and improving the prognosis of patients^45^. However, in a randomized controlled open-label trial involving hospitalized adult patients with confirmed SARS-CoV-2 infection, no benefit was observed with lopinavir-ritonavir treatment beyond standard care^46^. In the current study, the protective effect of tenofovir or protease-inhibitors remains inconclusive.

## Strengths and limitation

The strength of the current study is the global representation in the included articles assessing HIV/SARS-CoV-2 coinfection and estimated the attack and mortality rate in PLWHA compared to those who are HIV-negative. Thus, the findings from the current analysis are generalizable. Nevertheless, a lack of detailed study-level clinical and sociodemographic information did not allow us to perform subgroup and meta-regression analyses to explore the influence of stage of HIV, levels of CD4 counts or HIV viral load and ARV therapy regimen and treatment adherence on the incidence and severity of COVID-19 in HIV-positive persons. Furthermore, residual confounding by comorbid conditions cannot be excluded entirely. Therefore, future studies might consider assessing how the association between HIV and severe COVID-19 outcomes is moderated or mediated by comorbidities, HIV viral load, CD4 count, and ARV therapy. Lastly, although most studies only included laboratory-confirmed COVID-19 patients, a few studies combined laboratory-confirmed and clinically diagnosed (probable) cases. Therefore, it is possible that nondifferential misclassification of exposure could have affected the observed associations.

## Conclusion

HIV remains an important risk factor of acquiring SARS-CoV-2 infection and is associated with a higher risk of mortality from COVID-19. Therefore, PLWHA should be prioritized for receiving a vaccine. Further studies are warranted to assess the long-term outcomes in PLWHA who survives COVID-19.

## Supplementary Information

Supplementary Information.
